# Sex-related differences regarding headache triggered by low barometric pressure in Japan

**DOI:** 10.1186/s13104-024-06827-3

**Published:** 2024-07-23

**Authors:** Takuma Fujimoto, Hiroki Iwata, Noriko Kobayashi, Shingo Kondo, Katsunori Yamaura

**Affiliations:** 1 Division of Social Pharmacy, Center for Social Pharmacy and Pharmaceutical Care Sciences, Faculty of Pharmacy, Keio University, 1-5-30 Shibakoen, Minato-ku, Tokyo, 105-8512 Japan; 2 Keio University Community Pharmacy, Tokyo, Japan

**Keywords:** Headache, Headache triggering, Low barometric pressure, HIT-6, Sex difference

## Abstract

**Purpose:**

The prevalence of migraine headache is higher in women. Low barometric pressure is a factor in headache triggering, but sex-related differences have not been identified. The purpose of this study was to examine sex-related differences in headache triggered by low barometric pressure.

**Methods:**

Study subjects aged 20–49 years were randomly selected from a research company’s (Macromill, Inc.) web panel. Those with chronic migraine or tension-type headache invited to complete a web-based self-administered questionnaire. Logistic regression analysis was performed with the objective variable as the Headache Impact Test-6 (HIT-6) high scores (56 or more) or headache triggered by low barometric pressure.

**Results:**

Participants were 332 women and 337 men in the headache population. HIT-6 high scores were associated with age at headache occurrence 20 years or younger (OR: odds ratio 1.85, 95% CI: confidence interval 1.15–2.99, *p* = 0.012) and headache triggered by low barometric pressure (OR 2.11, 95%CI 1.51–2.94, *p* < 0.001). Headache triggered by low barometric pressure was significantly associated with women (OR 2.92, 95%CI 2.12–4.02, *p* < 0.001).

**Conclusions:**

Headache triggered by low barometric pressure were related to sex-related differences. It was suggested that a sex-specific treatment approach for headache triggering is needed.

**Supplementary Information:**

The online version contains supplementary material available at 10.1186/s13104-024-06827-3.

## Introduction

Sex differences are not only linked to biological factors, but also have an impact on quality of life (QoL). Sex-related differences in QoL can be attributed mainly to environmental factors and responses to illness. Clinical data have shown that there are sex-related differences in drug safety and efficacy [1], and the causes of these differences are now being investigated in terms of pharmacokinetics and pharmacodynamics [2], immunology [3], and genetics [4]. Biological sex-related differences have a non-negligible effect on pharmacokinetics. There is evidence suggesting that there are sex-related pharmacokinetic differences in drug absorption, distribution, metabolism, and excretion [5, 6]. However, the mechanisms underlying these biological differences between women and men remain unclear.

Headaches are a widespread and costly public health problem [7–9]. Studies have found that migraine is 3.3–4.4 times more common in women than in men and that tension-type headache is 1.5 times more common in women [10, 11]. Moreover, the prevalence of medication overuse headache (MOH) has been reported to be approximately 4 times higher in women than in men [12]. In terms of sex-related differences in the clinical features of migraine, women report a longer attack duration, increased likelihood of recurrence of headache, greater disability, and a longer recovery time [13].

Headache triggers include stress, female hormones, not eating, weather, sleep disturbance, and alcohol consumption [14], although it is unclear whether there are any sex-related differences in the overall risk profile. Low barometric pressure, barometric pressure changes, higher humidity, and rainfall have found to be associated with an increased number of headache occurrences [15–17]. People with migraine may be very sensitive to wet and cold stimuli, such as humidity and rainfall, and these environmental stresses may stimulate or modify the hypothalamus, triggering migraine attacks via the hypothalamus [16]. There are no studies have examined the factor of sex-related differences for headache triggered by low barometric pressure. Clarification of sex-related differences in headache triggering could lead to more effective treatment and self-care approaches.

The Headache Impact Test-6 (HIT-6) is a widely used measure for evaluating the impact of headache on QoL in daily life [18, 19]. The HIT-6 scores are not diagnostic but has the advantage of being able to self-check for headache in a short period of time. Although the HIT-6 high scores (56 scores or more) were used to determine the degree of disruption of daily life due to headache, there were no evidence of associated factors with HIT-6 high scores. It is possible that age at first headache occurrence may influence HIT-6 high scores, but previous studies have not investigated age at first headache occurrence. Clarification of the association with HIT-6 high scores, taking into account the factor of sex differences, will lead to effective treatment approaches.

In this study, we examine sex-related differences in headache triggered by low barometric pressure and effective factors associated with HIT-6 high scores.

## Methods

### Participants and study design

A web-based self-administered questionnaire survey was conducted on January 26 and 27, 2022. Aged 20–49 years were randomly selected from a web-panel at Macromill, Inc. The questionnaire used in this study was developed for this study [Supplementary]. Individuals older than 50 years were excluded because of the increased risk of headache occurring secondary to another illness [20], and individuals younger than 20 years old were also excluded. Those with chronic migraine or tension-type headache was selected by prescreening. Prescreening was conducted to ensure that individuals with headache that was neither tension-type nor migraine, those who had not experienced headache within the previous 3 months, and whose who were health care professionals were excluded. The survey was closed when 110 women and 110 men each in their 20s, 30s, and 40s had returned completed questionnaires. This survey included the 14-Item Health Literacy Scale (HLS-14), a useful tool for assessing cognitive and social skills underlying the motivation and ability of individuals to gain access to, understand, and use information in ways that promote and maintain good health [21]. The main items investigated were sex, age, HIT-6 scores, HLS-14 scores, headache type (tension-type, migraine), headache triggered by low barometric pressure, age at first headache occurrence, use of OTC or prescription drugs for headache, acute or prophylactic headache medication (unable to distinguish OTC or prescription), current using a headache app. Table 1 shows the definitions of tension-type or migraine headache, headache triggered by low barometric pressure and the age of first headache occurrence in this survey.

**Table 1:** Key questions in questionnaires

No. 1. Which of the following describes your headache? (Multiple choice) □ Tension-type headache: Tightening pain in the head and neck □ Migraine headache: Pulsating pain on one or both sides of the head □ Other headaches (free answer allowed) □ Don’t know [“Tension-type headache” or “Migraine headache” required to proceed to the main survey]
No. 2. When does your headache occur? (Multiple choice) □ Lack of sleep □ Excessive sleep □ Stiff shoulders and neck □ Physical overwork □ Tired eyes □ Mental stress □ When low barometric pressure approaches □ Change of seasons □ Other (free answer possible)
No. 3. At what age did you start having recurrent headaches? □ Input age □ Don’t know

### Statistical analysis

The HIT-6 ranges from 36 to 78, with higher scores indicating a worse impact of headache on daily life [17]. The HLS-14 ranges from 14 to 70, with higher scores indicating better health literacy [21]. Pearson’s chi-squared test, and Student’s two-sided *t*-test were used to assess differences between women and men in the headache population.

Logistic regression analysis was performed for scores of ≥ 56 on the HIT-6 as the objective variable, which indicates a substantial impact on daily life [17, 18]. Explanatory variables in our logistic regression model included women, Age 30–40 s (vs. 20s), first headache 20 years or younger, headache triggered by low barometric pressure, sleep duration under 5 h or over 8 h (vs. 5–8 h), HLS-14 52 or more. The HLS-14 scores were divided based on median scores of 52. Additionally logistic regression analysis was performed for headache triggered of low barometric pressure as the objective variable. Explanatory variables in our logistic regression model included women, Age 30–40 s (vs. 20s), first headache 20 years or younger, sleep duration under 5 h or over 8 h (vs. 5–8 h), HLS-14 52 or more. HIT-6 high scores were not included as an explanatory variable in the model of headache triggered by low barometric pressure because low barometric pressure is an exacerbating factor for headache [14]. Statistical analysis was performed using JMP Pro statistical software version 17.0.0 (JMP Statistical Discovery LLC, Cary, NC). A *p*-value < 0.05 was considered statistically significant.

## Results

### Sex differences in characteristics of participants

**Fig. 1:**
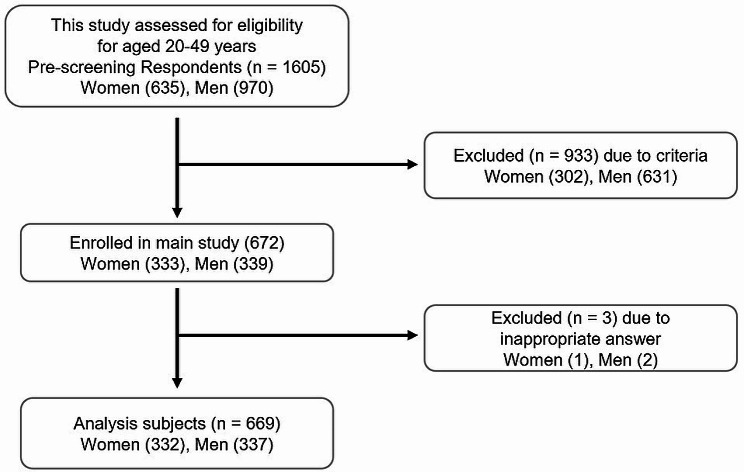
Flowchart describing questionnaire respondents selection. The study population was selected by prescreening of individuals with chronic tension-type or migraine headache. Subjects were the registered member at Macromill, Inc. Three respondents who chose the first check box for all questions were considered to have not answered the questionnaire items appropriately and were excluded

A flow chart of participants and exclusion criteria for this study is shown in Fig. 1. Individuals who met the age criterion of 20–49 years underwent prescreening. A total of 672 individuals (333 women, 339 men) were enrolled. After 3 exclusions for inappropriate responses, there were 669 participants (332 women, 337 men) for data analysis.

Table 2 shows the characteristics of the participants. In this study, there was no significant difference in the proportions of women and men who experienced migraine headaches (Table 1). Compared with men, women were significantly more likely to have HIT-6 high scores (56 or more) (*p* = 0.011), tension-type headache (*p* = 0.015), headache triggered by low barometric pressure (*p* < 0.001), use of OTC headache drugs (*p* < 0.001), acute headache medication (*p* < 0.001). Sleep duration was significantly different between women and men (*p* = 0.033).

**Table 2:** Characteristics of participants

	Women, *n* (%)	Men, *n* (%)	*p*-value
Patients, n	332 (100)	337 (100)	
Age, years			
20–29	112 (33.7)	110 (32.6)	NA
30–39	109 (32.8)	116 (34.4)	NA
40–49	111 (33.4)	111 (32.9)	NA
HIT-6 high scores (56 or more)	190 (54.0)	162 (46.0)	0.011*
Headache type			
Tension-type	132 (39.8)	103 (30.6)	0.015*
Migraine	284 (85.5)	289 (85.8)	0.932
Headache triggered by low barometric pressure	199 (59.9)	112 (33.2)	< 0.001***
Sleep duration under 5 h	37 (11.1)	36 (10.7)	0.033*
Sleep duration 5–8 h	269 (81.0)	290 (86.1)	
Sleep duration over 8 h	26 (7.8)	11 (3.3)	
Prescription headache drugs	57 (17.2)	41 (12.2)	0.083
OTC headache drugs	208 (62.7)	145 (43.0)	< 0.001***
Combination of prescription and OTC drugs	20 (6.0)	11 (3.3)	0.088
Acute headache medication	221 (66.6)	159 (47.2)	< 0.001***
Prophylactic headache medication	4 (1.2)	10 (3.0)	0.111
Combination of acute and prophylactic headache medication	3 (0.9)	10 (3.0)	0.053
Headache apps current users	24 (7.2)	16 (4.7)	0.176

Data are shown as the number and percentage according to sex. *p*-values were obtained using Pearson’s chi-squared test. NA, not available; OTC, over-the-counter

### Factors in association with HIT-6 high scores

**Fig. 2:**
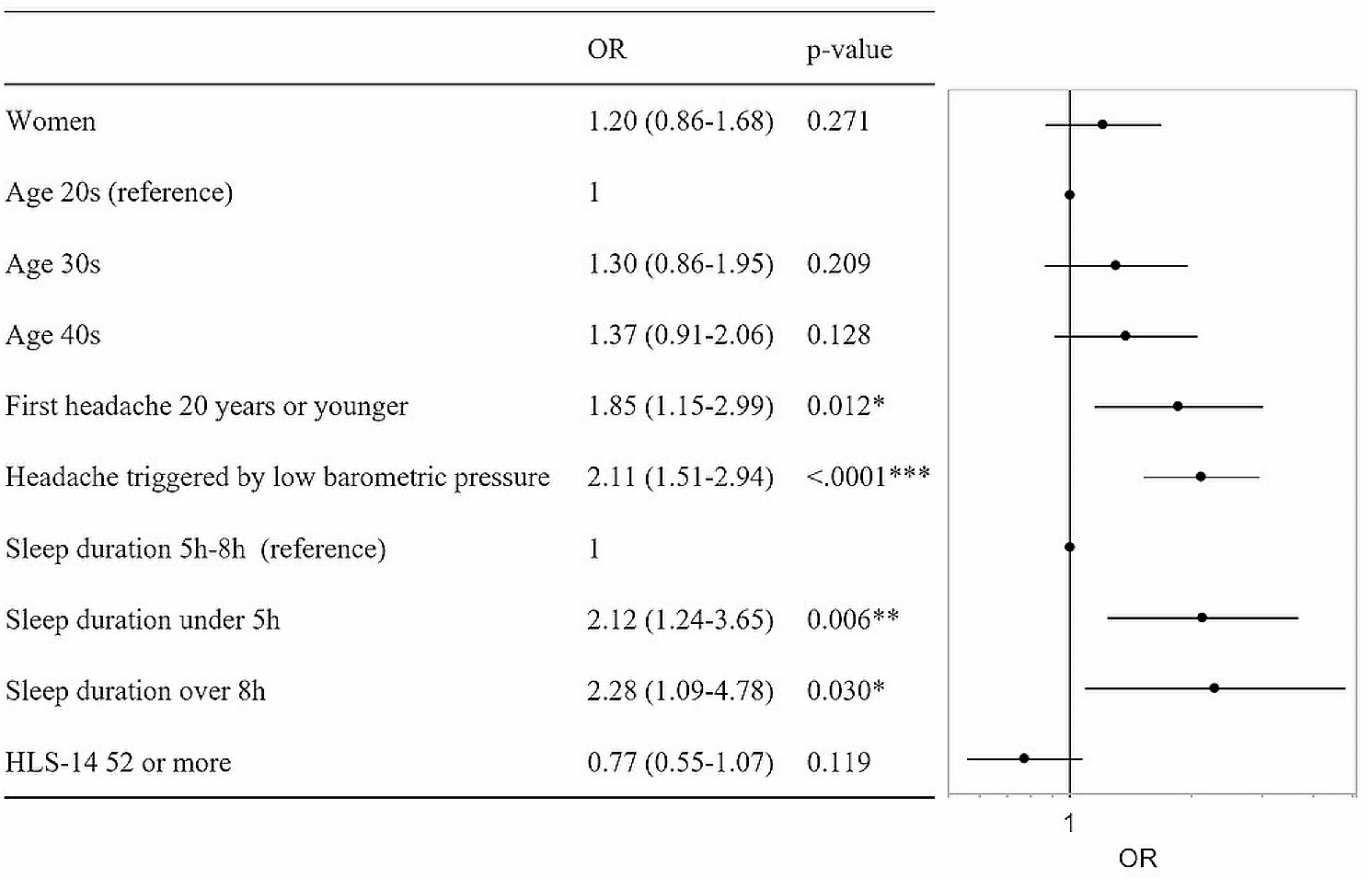
Variables associated with higher HIT-6 scores in logistic regression analysis. The attributes of individuals with HIT-6 scores of ≥ 56 are presented as the adjusted OR and 95% CI. Reference categories for calculating odds ratios were men, age in 20s, first headache occurred in 20 years old or younger, headache not triggered by low barometric pressure, sleep duration of 5–8 h, and HLS-14 ≤ 51. *p < 0.05, **p < 0.01, ***p < 0.001. CI, confidence interval; OR, odds ratio

HIT-6 high scores were not significantly associated with sex-related difference. HIT-6 high scores were significantly associated with age at headache occurrence 20 years or younger (OR: odds ratio 1.85, 95% CI: confidence interval 1.15–2.99, *p* = 0.012), headache triggered by low barometric pressure (OR 2.11, 95%CI 1.51–2.94, *p* < 0.001), sleep duration under 5 h (OR 2.12, 95%CI 1.24–3.65, *p* = 0.006), sleep duration over 8 h (OR 2.28, 95%CI 1.09–4.78, *p* = 0.030) (Fig. 2.).

### Factors in association with headache triggered by low barometric pressure

**Fig. 3:**
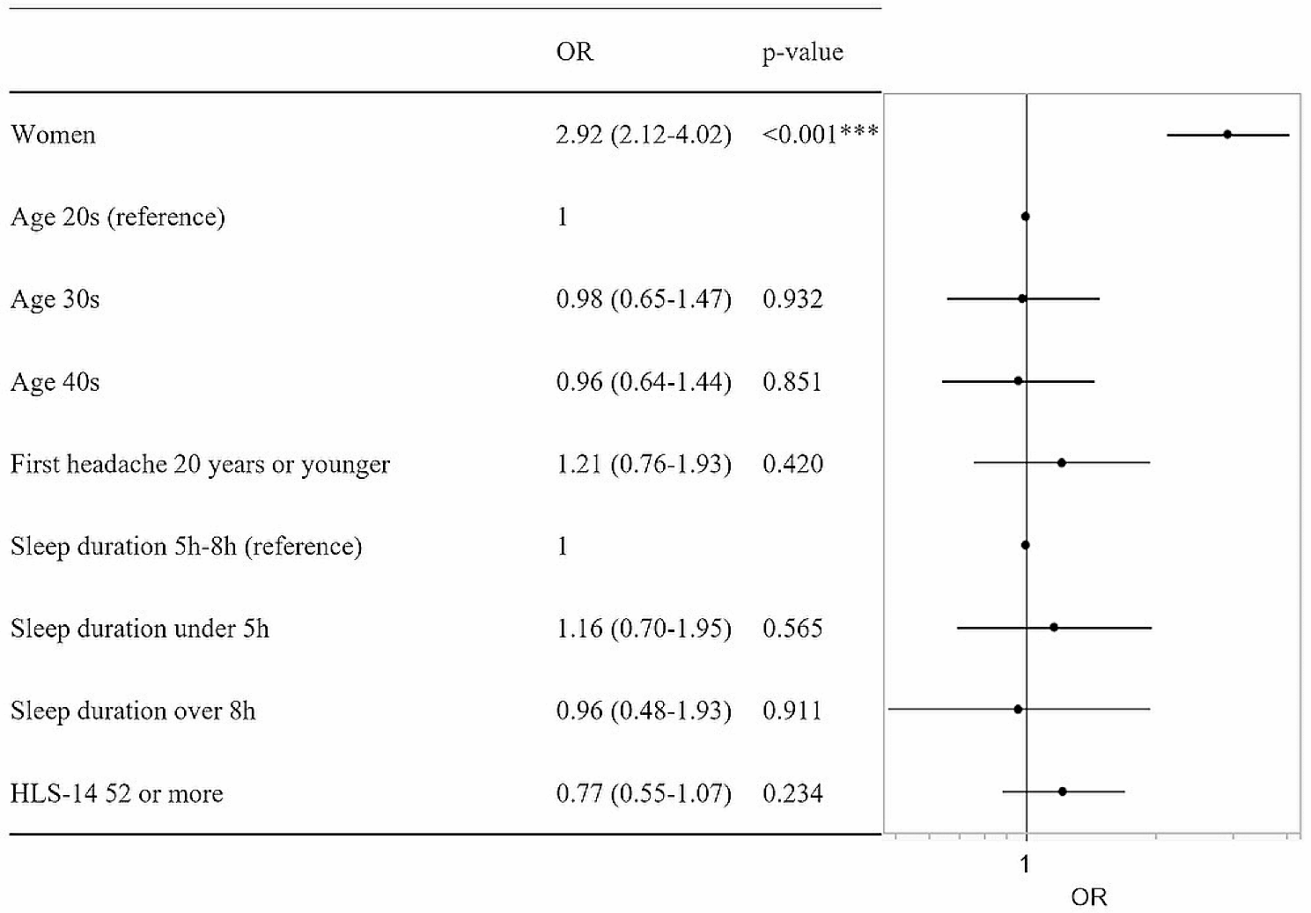
Variables associated with headache triggered by low barometric pressure in logistic regression analysis. The attributes of individuals with headache triggered by low barometric pressure are presented as the adjusted OR and 95% CI. Reference categories for calculating odds ratios were men, age in 20s, first headache occurred in 20 years old or younger, sleep duration of 5–8 h, and HLS-14 ≤ 51. *p < 0.05, **p < 0.01, ***p < 0.001. CI, confidence interval; OR, odds ratio

Headache triggered by low barometric pressure was significantly associated with women (OR 2.92, 95%CI 2.12–4.02, *p* < 0.001) (Fig. 3.).

### Sex differences in age at first headache occurrence

We found that women experienced their first headache at a younger age than men (23.0 ± 8.8 years vs. 25.1 ± 9.1 years; *p* = 0.026; Table 3).

**Table 3:** Sex-related differences in the age at first headache occurrence

	Women (*n* = 164)	Men (*n* = 178)	*p*-value
Age, years ± standard division	23.0 ± 8.8	25.1 ± 9.1	0.026*

The denominator is different here because those who answered “I don’t know” about the age at first headache occurrence were excluded

## Discussion

This study aimed to investigate sex-related difference in factors associated with headache. Headache triggered by low barometric pressure were related to sex-related differences (Fig. 3). On the other hand, HIT-6 high scores were not significantly associated with sex-related differences (Fig. 2). Additionally, the age first headache occurred in women was significantly younger than in men (Table 3). Thus, it was suggested that a sex-specific treatment approach for headache triggering is needed.

Prior studies have indicated that low barometric pressure is a trigger of headache [14–17]. Sato J, et al. showed that lowering barometric pressure induces neuronal activation in the superior vestibular nucleus in mice [22]. This study showed that headache triggered by low barometric pressure were related to sex-related differences (Fig. 3). Therefore, it is necessary to examine sex-related differences of neuronal activation in the superior vestibular nucleus.

This study is the first to show a sex difference in age at first headache occurrence (Table 3) and its association with HIT-6 high scores (Fig. 3). The association between age at headache occurrence has not received much attention. Age at headache onset may be related to QoL due to headache. This study suggests the need for communication with patients considering the age of headache occurrence.

62.7% of women used headache medication OTC. This was significantly higher than the 43.0% of males (Table 2). Recording medication intake using a headache diary or headache app is useful for monitoring symptoms. In Japan, the Okusuri-techo handbook, which records drug prescriptions and provides information on medicines, is often used for this purpose. Information on OTC drugs is also being considered for inclusion in the electronic version of the medication handbook [23]. Additionally, in Japan, high school students who abuse OTC drugs are more likely to be women than men [24], and the scope of OTC drugs with potential for abuse was expanded in 2023 [25]. Inadequate knowledge about OTC drugs may lead to medication overuse headache, MOH [26]. Keeping in mind the sex differences in headache medication OTC may lead to more appropriate drug use, including awareness of the risk of overdose.

### Limitations

The strength of this study is that it has identified sex-related differences in headache triggered by low barometric pressure with logistic regression analysis. However, this study also has some limitations. First, headache triggered by low barometric pressure is based on self-report and not on smartphone records and actual weather conditions. Second, this study did not investigate OTC for headaches increased due to the headaches experienced during low barometric pressure. A previous study shows that sales of OTC loxoprofen, which can represent the onset and aggravation of headache, significantly increased with worsening weather conditions [27]. Third, this study did not include taking anti-contraceptives by women. Anti-contraceptives can play a role as it directly relates to female hormones. Forth, cold hypersensitivity (Hie or Hiesho in Japanese) can cause distress and hinder the execution of routine activities [28, 29]. In Japan, women with cold hypersensitivity report a painful cold sensation and associated symptoms such as insomnia, fatigue, and edema [30]. Further, cold hypersensitivity is associated with developing chronic conditions, such as dysmenorrhea, rheumatic diseases, migraines, and vascular diseases [31, 32]. Also, cold hypersensitivity is associated with frailty [33]. Therefore, understanding the association between cold hypersensitivity and headache is essential for effective management and treatment. Fifth, analgesics are used not only for headache but also for other types of pain, including menstruation-related pain and low back pain. Furthermore, our survey did not include the frequency of use of drugs for headache, which is one of the diagnostic criteria for MOH in the third edition of the International Classification of Headache Disorders [34]. Examining sex-related differences in the relationship of the purpose and frequency of analgesic use with headache can highlight issues related to the appropriate use of analgesics. Sixth, this study did not include loneliness. In a previous study, it was shown that increased loneliness and the severity of the perceived social isolation is associated with the prevalence/incidence of pain [35]. Thus, additional factors of cold hypersensitivity and frequency of analgesic use should be considered with regard to sex-related differences in headache.

### Electronic supplementary material

Below is the link to the electronic supplementary material.

Supplementary Material 1

## Data Availability

No datasets were generated or analysed during the current study.
