# Supplementary material for: Sex-related differences regarding headache triggered by low barometric pressure in Japan
Source: BMC Res Notes. 2024 Jul 23;17:203. doi: 10.1186/s13104-024-06827-3 (PMC11267689; doi:10.1186/s13104-024-06827-3)
Supplement: Supplementary file 1 — Supplementary Material 1 [file 13104_2024_6827_MOESM1_ESM.pdf]

Questionnaire

[Note: the text in square brackets is presented here to show the flow of the survey and was not displayed during the actual survey.]

[Screening questions]

No. 1. Are you a health care professional? (Single answer)
□ Yes [“No” required to proceed to the main survey]
□ No

No. 2. Have you had any headaches in the past 3 months? (Single answer)
□ Yes [“Yes” required to proceed to the main survey]
□ No

No. 3. Which of the following describes your headache? (Multiple choice)
□ Tension-type headache: Tightening pain in the head and neck
□ Migraine headache: Pulsating pain on one or both sides of the head
□ Other headaches (free answer allowed)
□ Don't know
[“Tension-type headache” or “Migraine headache” required to proceed to the main survey]

No. 4. How many times a year do you have headaches?
□ 1~3 times a year (less than once every 4 months)
□ 4~6 times a year (once every 2~3 months)
□ 7~12 times a year (once every 1~2 months)
□ More than 12 times a year (more than once a month)
[More than 3 times a year required to proceed to the main survey]

No. 5. How many days in the past 3 months have you had headaches? Please enter the approximate number of days even if you do not have an exact memory.
□ Select the number of days [1 day or more required to proceed to the main survey]

[Respondents who were excluded based on the above screening questions were not shown the main survey.]

[Main survey]

No. 6. When does your headache occur? (Multiple choice)
□ Lack of sleep
□ Excessive sleep
□ Stiff shoulders and neck
□ Physical overwork
□ Tired eyes
□ Mental stress
□ When low barometric pressure approaches
□ Change of seasons
□ Other (free answer possible)

No. 7. At what age did you start having recurrent headaches?

□ Input age
□ I don't know

No. 8. About headache applications focused on weather forecasting (barometric pressure changes)

□ I don't know
□ Have never used it
□ Have used, but not currently using
□ Currently using

<u>Headache Impact Test (HIT-6: Headache Impact Test-6)</u>

[The HIT-6 from the following work was administered.

Yang M, Rendas-Baum R, Varon SF, Kosinski M. Validation of the Headache Impact Test (HIT-6) across episodic and chronic migraine. Cephalalgia. 2011;31(3):357-367. doi:10.1177/0333102410379890.]

<u>Lifestyle related to sleep</u>

No. 9. Over the past month, what has been your average amount of sleep per day? (Single answer)

□ Less than 5 hours
□ 5 hours or more but less than 6 hours
□ 6 hours or more but less than 7 hours
□ 7 hours or more but less than 8 hours
□ 8 hours or more but less than 9 hours
□ More than 9 hours

<u>Use of headache medication</u>

No. 10. Do you currently use over-the-counter medications for headaches?

□ Yes
□ No
□ I don’t know

No. 11. Do you currently use prescription medications for headaches?

□ Yes
□ No
□ I don’t know

No. 12. What is the name of the medication you are currently using for headaches? Please select the appropriate option(s). (Multiple answers allowed)

□ Loxoprofen(Loxonin) □ Ibuprofen □ Acetaminophen(Kalonal) □ Sumatriptan(Imigran)
□ Zolmitriptan(Zomic) □ Lomerizine Hydrochloride (MIGSYS) □ Valproate □ Topiramate (Topina)
□ Propranolol Hydrochloride (Inderal) □ Amitriptyline
□ Kampo Goshuyu-tou □ Kampo Goreizan
□ Antibody drug galcanezumab (Emgality)
□ Antibody drug elenumab (aimovig)
□ Antibody drug furemanezumab (AJOVY)
□ Antibody drug (any of the above or other antibody drugs)
□ Other (Free answer)
□ I don't know

Health Literacy: 14-item Health literacy scale (HLS-14)

[The HLS-14 from the following work was administered.

Suka M, Odajima T, Kasai M, Igarashi A, Ishikawa H, Kusama M, Nakayama T, Sumitani M, Sugimori H. The 14-item health literacy scale for Japanese adults (HLS-14). Environ Health Prev Med. 2013;18(5):407-15. doi: 10.1007/s12199-013-0340-z. Epub 2013 May 21.]

This concludes this survey. Thank you very much for your cooperation.
